# Sex Hormone-Binding Globulin (SHBG) Expression in Ovarian Carcinomas and Its Clinicopathological Associations

**DOI:** 10.1371/journal.pone.0083238

**Published:** 2013-12-26

**Authors:** Ruixia Huang, Yuanyuan Ma, Ruth Holm, Claes G. Trope, Jahn M. Nesland, Zhenhe Suo

**Affiliations:** 1 Departments of Pathology, The Norwegian Radium Hospital, Oslo University Hospital, Oslo, Norway; 2 Departments of Pathology, Institute of Clinical Medicine, Faculty of Medicine, University of Oslo, Oslo, Norway; 3 Departments of Gynecology, The Norwegian Radium Hospital, Oslo University Hospital, Oslo, Norway; 4 Departments of Gynecology, Institute of Clinical Medicine, Faculty of Medicine, University of Oslo, Oslo, Norway; Mayo Clinic College of Medicine, United States of America

## Abstract

Sex hormone-binding globulin (SHBG) is known as a carrier protein. It is classically thought to be mainly synthesized in the liver and then secreted into the circulating system, where it binds to sex steroids with a high affinity and modulates the bio-availability of the hormones. Other organs known to produce SHBG include brain, uterus, testis, prostate, breast and ovary, and the local expressed SHBG may play an important role in tumor development. However, SHBG expression status and its clinicopathological significance in ovarian cancer cells are not reported yet. In our present study, we examined and found the variable SHBG expression in four ovarian cancer cell lines (OV-90, OVCAR-3, SKOV-3 and ES-2) by immunocytochemistry and Western blotting. We then extended our study to 248 ovarian carcinoma samples, which were collected at The Norwegian Radium Hospital, Oslo University Hospital with complete clinical information, and discovered that SHBG was variably expressed in these ovarian carcinomas. Higher level of SHBG expression was significantly associated with more aggressive histological subtype (*p* = 0.022), higher FIGO stage (*p* = 0.018) and higher histological grade (grade of differentiation, *p* = 0.020), although association between SHBG expression and OS/PFS was not observed. Our results demonstrate that ovarian cancer cells produce SHBG and higher SHBG expression in ovarian carcinoma is associated with unfavorable clinicopathological features.

## Introduction

Ovarian carcinoma is the third commonest malignancy with the most mortality in gynecologic tumors [1], [2]. Hormonal stimulation of ovarian epithelial cells is suggested as a mechanism for carcinogenesis of the ovary [3], [4]. Sex hormone-binding globulin (SHBG), a 373-amino-acid glycoprotein, known as a carrier protein before, is classically thought to be mainly synthesized in the liver and then secreted into the circulating system [5], [6], [7], where it binds to sex steroids with a high affinity and modulates the bio-availability of the hormones, such as estrogen, androgen and testosterone [4], [8], [9]. There are reports that plasma SHBG may play a role in the development of several sex hormone-related carcinomas, e.g. breast [10], prostate [11] and ovarian [12] carcinomas. Circulating SHBG might contribute to the ovarian carcinogenesis through regulation of sex hormone bio-availability [4], [9], [13], [14].

Besides liver cells, SHBG is also produced in other organs, including brain [15], [16], uterus [17], testis [18], [19], prostate [20], [21], breast [20], ovary [22] and placenta [23], etc. Moreover, the locally expressed SHBG may participate in the membrane-based steroid signaling pathway and modulate the androgen receptor (AR) and estrogen receptor (ER) activations in the prostate and breast cancer cells, thus it may play a functional role in the steroid responsiveness of prostate and breast cells and the perturbation of local SHBG expression could contribute to prostate and breast cancers [20]. The local expression of SHBG in human ovarian carcinoma cells and its clinicopathological correlations are currently unknown.

In our present study, we have investigated the expression of SHBG in four ovarian cancer cell lines by immunocytochemistry (ICC) and Western blotting, verifying variable SHBG expression in these cell lines. Then we extended this study to 248 ovarian carcinoma patients, in order to explore whether SHBG is expressed in the human ovarian carcinomas and furthermore analyze the associations between the local SHBG expression and clinicopathological features, including age, histological subtype, histological grade (grade of differentiation), International Federation of Gynecology and Obstetrics stage (FIGO stage), overall survival (OS) and progression free survival (PFS).

## Materials and Methods

### Ethics Statement

The Regional Committee for Medical Research Ethics South of Norway (S-06277a), The Social- and Health Directorate (06/3280) and The Data Inspectorate (06/5345) approved the study. All the patients involved provided their written consent to participate in this study, and all the written consents were filed in The Norwegian Radium Hospital, Oslo University Hospital.

### Cell Lines

Four ovarian cancer cell lines ES-2, SKOV-3, OVCAR-3 and OV-90 (from American Type Culture Collection, USA) were maintained in our laboratory. The ES-2 line was derived from a patient with ovarian clear cell carcinoma (CCC), and other three cell lines were derived from malignant ascites of ovarian adenocarcinoma patients. All cells were cultivated in PRMI 1640 medium supplemented with 10% fetal bovine serum (FBS), 100units/ml penicillin and 100 µg/ml streptomycin at 37°C in a humidified 5% CO_2_ incubator.

### Immunocytochemistry (ICC)

Cytoblocks were prepared for ICC. For each cell line, the cells in 80% confluent were harvested by mechanical scraping, and cells in suspension were spun down at 2000 rpm for 5 minutes before the supernatant was discarded. The cells were rinsed twice with phosphate-buffered saline (PBS) to further delete the dead cells or cell organelles. Four drops of plasma and 2 drops of thrombin were added to the sedimentation after the supernatant was discarded, and the contents were carefully mixed by rotating tube for one minute before coagulation was formed. 4% buffered formaldehyde was added to the coagulation for cell fixation. The coagulated mass was then wrapped in a linen paper, put in a labeled cassette, and placed in 4% buffered formaldehyde. The material was paraffin-embedded to make cytoblock before being cut into 3 µm paraffin sections for ICC.

Dako Envision™ FLEX+ system (Dako, Denmark) and the Dako Autostainer were used for ICC. Paraffin sections were deparaffinized and epitopes were unmasked in PT link with low pH target retrieval solution (Dako), and then blocked with peroxidase blocking (Dako) for 5 minutes. The slides were incubated overnight at 4°C with goat anti-human SHBG antibody (1∶1200, 0.17 µg IgG/ml, cat no. AF2656, R&D,). The secondary antibody (1∶100, mouse anti-goat IgG, Santa Cruz Biotechnology) was incubated at room temperature for 30 minutes, followed with mouse linker for 15 minutes and HRP for 30 minutes at room temperature. Slides were then stained with 3, 3′-diaminobenzidine tetrahydrochloride (DAB, Dako) for 10 minutes and counter-stained with hematoxylin, dehydrated, and mounted in Richard-Allan Scientific Cyto seal XYL (Thermo Scientific, Waltham, MA, USA). Known SHBG-positive human liver tissue was used as positive control in the same procedure as for ovarian cancer cell lines or carcinoma slides. The corresponding non-immune goat IgG was used as negative control at the same concentration as the primary goat anti-human SHBG antibody.

Intensity scoring was used to evaluate the staining level of SHBG in the slides, considering the rather homogeneous staining. The results were separated into four groups: score 0 standing for negative, while score 1, 2 and 3 representing weakly, moderately, and strongly stained, respectively.

### Western Blotting

For each ovarian carcinoma cell line, the cells were digested by 0.25% trypsin and EDTA (Invitrogen) when cells grew 80% confluent and cells in suspension were spun down at 1200 rpm for 5 minutes before supernatant was discarded. After washed with ice-cold PBS twice, the cells were dissolved with lysis buffer containing RIPA buffer (Thermo scientific), 1% PMSF, 1% aprotinin, 1% leupeptin, 1% pepstatin and 0.5% vanadate by pipetting gently up and down, put on ice before spun down at 14000 rpm for 15 minutes at 4°C to release total protein in the supernatant. Total protein was measured by the Bio-Rad protein assay (Hercules, CA, USA). Equal amount of proteins from each sample in SDS loading buffer was boiled for 5 minutes and subjected to 10% SDS-PAGE electrophoresis and then electro-transferred to high-quality polyvinylidene difluoride (PVDF) membrane in a Trans-Blot apparatus (Bio-rad, Hercules, CA). The membrane was blocked with 5% fat-free milk for 1 hour at room temperature and incubated overnight at 4°C with goat anti-human SHBG antibody (1∶500, 0.4 µg/ml, cat. no. AF2656, R&D). After washing with PBS-tween 0.05% (PBST), the blot was incubated with rabbit anti-goat IgG HRP antibody for 45 minutes at room temperature (1∶1000, cat. no. HAF017, R&D). After several washes with PBST, the blot was visualized using an enhanced chemiluminescence detection kit (ECL, Amersham) by following the manual guide.

### Clinical Samples

Two hundred and forty-eight surgically removed ovarian carcinoma samples were enrolled in this study. All patients were operated at The Norwegian Radium Hospital, Oslo University Hospital from March 1983 to May 2001. The ages of the patients range from 19 to 89 years, with a median of 58 years. The patients were followed up until January 1^st^ 2012. All the patients were clinically staged by FIGO stage method [24]. The primary tumors were histologically graded as well, moderately and poorly differentiated according to WHO recommendations [24]. Disease progression was determined based on the definitions outlined by the Gynecologic Cancer Intergroup [25]. Paraffin-embedded ovarian carcinoma tissues were obtained from the file of the Department of Pathology, and 3 µm sections were cut and used for morphological examination and immunohistochemistry (IHC) with the same procedure as for ICC.

### Statistical Analyses

SPSS software (version 18.0) was used for data analysis. Associations between categorical variables were assessed by Chi-square tests (Pearson and linear-by-linear as appropriate). Survival analysis was estimated using the Kaplan-Meier method, and groups were compared with log-rank tests. For all the analyses, associations were considered to be significant if the *p* value was <0.05.

## Results

### Expression of SHBG in Ovarian Cancer Cell Lines

**Figure 1:**
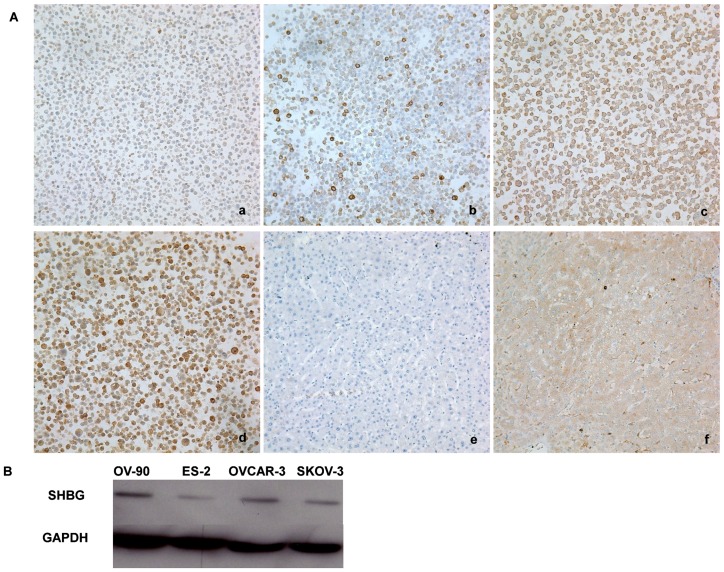
SHBG expression in ovarian cancer cell lines. **A.** ICC. (a): weak SHBG immunodetection in ES-2 cell line; (b): moderate SHBG immunodetection in SKOV-3 cell line; (c) and (d): strong SHBG immunodetection in OVCAR-3 cell line and OV-90 cell line, respectively; (e): negative human liver tissue control with the same concentration of non-immune goat IgG; (f): positive human liver tissue control showing positive cytoplasmic SHBG expression. All the photos were taken at 200×. **B.** Western blotting. It shows similar expression levels of SHBG as with ICC result in the four ovarian cancer cell lines, i.e.: high expression in OV-90 and OVCAR-3 cell lines, moderate expression in SKOV-3 cell line and weak expression in ES-2 cell line.

Cytoplasmic SHBG was detected by ICC in all of the four ovarian cancer cell lines ES-2, SKOV-3, OVCAR-3 and OV-90 (Fig. 1A a, b, c, d). Comparatively, it was discovered that ES-2 cell line was weakly positive (intensity score was 1, Fig. 1A a), SKOV-3 cell line was moderately positive (intensity score was 2, Fig. 1A b), and OVCAR-3 and OV-90 cell lines were strongly positive (intensity score was 3 for both, Fig. 1A c and Fig. 1A d, respectively) for SHBG expression.

The SHBG protein expression in these cell lines was confirmed by Western blotting as well. This assay revealed an immunoreactive band of 47 kDa (Fig. 1B), in line with the suggested molecular weight as reported by others [16], [19], [26]. Quantitative analysis of the blots revealed that the SHBG expressions in the four ovarian cancer cell lines agreed with the ICC finding: high in OV-90 and OVCAR-3, moderate in SKOV-3 and low in ES-2.

### SHBG Immunodetection in Clinical Ovarian Carcinoma Samples

**Figure 2:**
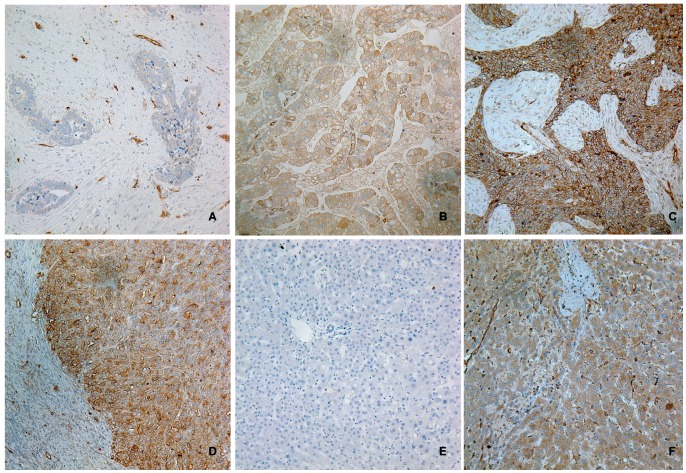
IHC SHBG staining in clinical ovarian carcinoma samples. Representative weak (A), moderate (B, moderately differentiated tumor) and strong (C, poorly differentiated tumor) are shown. Stronger SHBG immunostaining is shown at the invasive front of a poor-differentiated ovarian carcinoma (D). Negative control performed in human liver tissue with the same concentration of non-immune goat IgG shows no staining in the liver cells (E). Positive control performed with liver tissue shows the positive cytoplasm SHBG staining. Endothelial cells are also positive for SHBG. All photos were taken at 200×.

Immunoreactive SHBG was variably detected in the ovarian carcinoma cells in all the ovarian primary tumor samples (Fig. 2). The immunostaining was limited to cytoplasm and cell membrane of ovarian carcinoma cells, and the nuclei were never stained. In addition to the positive tumor cells, endothelial cells were also immunoreactive for SHBG.

The immunostaining level of SHBG was scored by the intensity scoring with the same criteria as for ICC, for it was also relatively homogenous in each sample. According to the IHC score, the 248 samples were divided into 3 groups: weak expression group (n = 65), moderate expression group (n = 93), and strong expression group (n = 90). In addition, carcinoma cells at the invasive front/infiltration front [27], [28] were more strongly stained than other tumor cells in the same primary tumor (Fig. 2d).

### Clinicopathological Associations

The associations of clinical features including age, histology, FIGO stage and grade of differentiation with SHBG expression in the ovarian carcinomas were shown in Table 1. SHBG expression was positively associated with histological subtype (*p* = 0.022), FIGO stage (*p* = 0.018) and histological grade (grade of differentiation, *p* = 0.020). For tumor histological subtype grouping, the rates of weakly positive SHBG in group 1 (serous tumor), group 2 (mucinous and endometrioid tumors) and group 3 (clear cell, mixed and undifferentiated tumors) were 25.2% (41/163), 45.9% (17/37) and 14.8% (4/27) respectively, and strong SHBG detection rates in these three groups were 41.1% (67/163), 18.9% (7/37) and 40.7% (11/27), respectively. As for FIGO stage, weakly positive SHBG expression rates in stage I/II, III and IV tumors were decreased from 34.8% (16/46), to 27.4% (32/117) and then to 21.5% (17/79), and strongly positive SHBG expression rates in these three groups were correspondingly increased from 23.9% (11/46) to 36.8% (43/117) and then to 45.6% (36/79), respectively. In consideration of histological grade, strong SHBG positivity from well, moderate to poor differentiated tumors was significantly increased, from 15.8% (3/19) to 29.0% (18/62) and then to 44.4% (59/133). Similarly, the percentage for tumors of weakly positive SHBG from well, moderate to poor differentiated tumors was correspondingly decreased. Linear-by-linear Chi-square test showed a significantly positive association between higher level of SHBG expression and poor histological grade (*p* = 0.020).

**Table 1:** Comparison of clinical and pathologic characteristics by tumor SHBG intensity.

		SHBG IHC score			
		1 (weak)	2 (moderate)	3 (strong)	*p* value
N	Total N	65	93	90	
Age (years old):					0.200
≤49	57	19	19	19	
		(33.3%)	(33.3%)	(33.3%)	
≥50	175	41	66	68	
		(23.4%)	(37.7%)	(38.9%)	
Missing	16				
Histological subtype:					0.022
Serous	163	41	55	67	
		(25.2%)	(33.7%)	(41.1%)	
Muci+Endo	37	17	13	7	
		(45.0%)	(35.1%)	(18.9%)	
Clea+Mixe+Undi	27	4	12	11	
		(14.8%)	(44.4%)	(40.7%)	
Others or unclassified	21				
FIGO Stage:					0.018
I+II	46	16	19	11	
		(34.8%)	(41.3%)	(23.9%)	
III	117	32	42	43	
		(27.4%)	(35.9%)	(36.8%)	
IV	79	17	26	36	
		(21.5%)	(32.9%)	(45.6%)	
Not staged or missing	6				
Histological Grade:					0.020
Well	19	6	10	3	
		(31.6%)	(52.6%)	(15.8%)	
Moderate	62	18	26	18	
		(29.0%)	(41.9%)	(29.0%)	
Poor	133	31	43	59	
		(23.3%)	(32.3%)	(44.4%)	
Not graded or missing	34				

Muci, Mucinous tumor; Endo, Endometrioid; Clea, Clear cell; Mixe, Mixed epithelial tumor; Undi, Undifferenciated tumor.

### Survival Analyses

**Figure 3:**
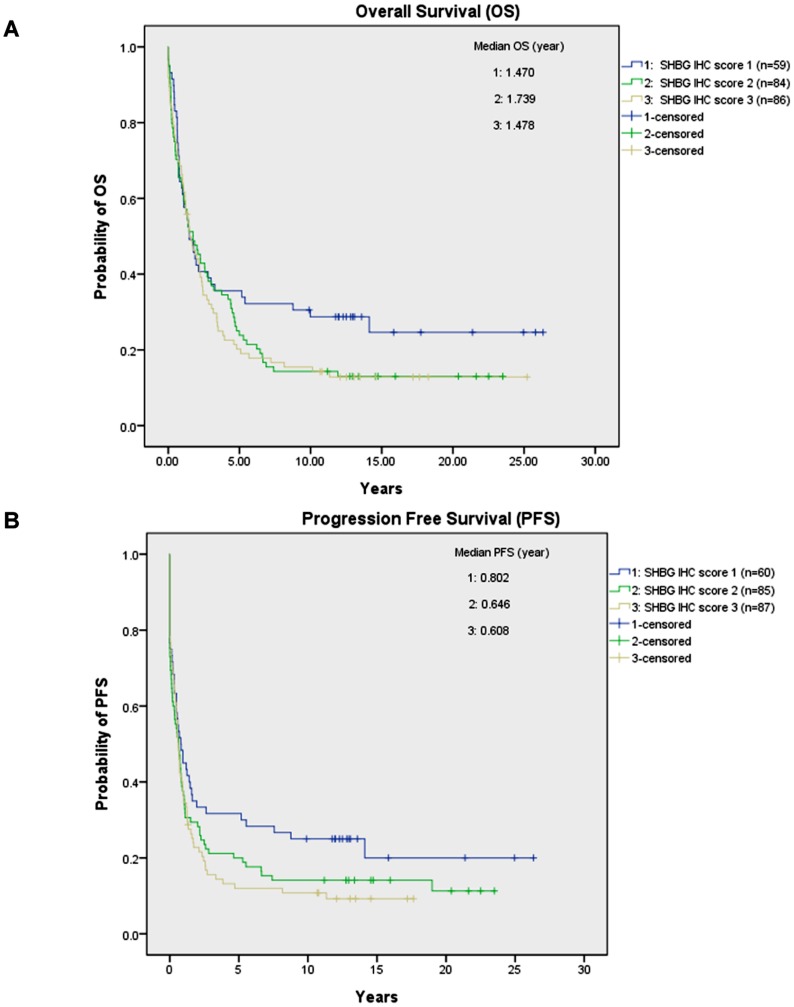
OS and PFS for the ovarian carcinoma patients in 3 SHBG IHC groups. For both OS (A) and PFS (B) curves, weakly (IHC score 1), moderately (IHC score 2) and strongly (IHC score 3) SHBG expressing groups were marked by blue, green and brown lines respectively. No statistically significant difference was obtained both for OS (*p* = 0.220) and PFS (*p* = 0.132).

Statistically there were no significant associations between SHBG expression and OS (*p* = 0.220) or PFS (*p* = 0.132), although there was a negative association trend (Fig. 3). The median OS for the 229 valid cases was 1.602 years with 95% confidence interval (CI) at 1.188 to 2.015 years. The median PFS for the 232 valid cases was 0.646 year with 95% CI at 0.501 to 0.791 year. The median PFSs in the SHBG weakly positive group, moderately positive group and strongly positive group were 0.802, 0.646, and 0.608 year respectively.

## Discussion

Both female and male sex hormones play important roles in ovarian carcinoma proliferation, invasiveness and metastasis [29], [30], [31]. SHBG, known as sex hormone carrier protein, is synthesized mainly in the liver and secreted into blood, regulating the free concentration of the steroids that bind to it in plasma [32], [33], [34]. Plasma SHBG is reduced by tumor necrosis factor-α (TNF-α) via downregulating hepatocyte nuclear factor 4-α (HNF4-α), a key transcription factor that regulates SHBG expression in the liver [35]. SHBG can also participate in multiple signaling pathways for certain steroid hormones on selected cell membranes. It binds with high affinity to a specific membrane receptor (R_SHBG_), forming the SHBG-R_SHBG_ complex [36], [37], and an appropriate steroid binds to this complex and increases intracellular cAMP in prostate and breast cancers [38], [39], [40]. As a result, plasma SHBG is assumed to play a role in ovarian carcinoma clinical behaviors. However, a case-control study of 132 ovarian carcinoma patients with primary ovarian carcinomas and 264 controls in parallel from New York (USA), Umea (Sweden) and Milan (Italy) did not reveal a strong relation between increasing circulating SHBG and ovarian carcinoma risk [41], also confirmed by Rinaldi and coworkers [42].

In the other hand, increasing number of studies confirms that SHBG production is not limited in liver cells, although the function of the locally synthesized SHBG in non-liver cells is not clear at present. Forges and coworkers [43] detected SHBG in human ovarian follicles and corpora lutea and indicated a significant role for SHBG in regulating follicular maturation as well as in luteal function. Intracellular expression of SHBG was detected in breast cancer cell line MCF-7 both in mRNA level by real-time PCR and protein level by Western blotting, and it was proved to be dose-dependent of estrogen in the cell culture medium [44]. Hryb and colleagues [21] investigated both SHBG mRNA and protein in prostate cancer cell lines (LNCaP, PC 3, and DU 145) as well as in cultured prostate stromal and epithelial cells derived from patients with benign prostatic hyperplasia by biochemical and immunocytochemical techniques, and reported locally synthesized SHBG in the human prostate with an autocrine/paracrine effect. The locally expressed SHBG was later reported to be involved in the regulation of the responsiveness of prostate and breast cells via multiple signaling pathways, suggesting its role in prostate and breast carcinomas [20], which was supported by a report of Nakhla et al. [45] stating that aberrant SHBG expression in hormonally responsive tissues might contribute to carcinogenesis.

Our present report is, to our best knowledge, the first study to systematically assess the localization and clinicopathological relations of the locally expressed SHBG in human ovarian carcinomas. We detected SHBG expression in all the four human ovarian cancer cell lines both by ICC and Western blotting, confirming the intracellular expression of SHBG in different histological subtype-derived cell lines. The OV-90 line which was derived from the ascites of a patient with histological grade 3/stage IIIC malignant papillary serous adenocarcinoma, expressed the highest level of SHBG. The ES-2 line, derived from an ovarian CCC with fibroblast morphology, expressed the lowest level of SHBG.

In our present study, we did find that SHBG was significantly highly expressed in the group of clear cell, mixed and undifferentiated tumors, compared to the serous and mucinous/endocrine groups. CCC in early stage (FIGO stage I) did reveal a favorable prognosis as stage I serous carcinoma (SC), however CCC in advanced stage (FIGO stage II, III and IV) was linked to a poor clinical result [46]. Moreover, it is commonly known that CCC is highly resistant to most chemotherapy regimens [46], [47]. However ES-2 line exhibits low to moderate resistance to a number of chemotherapeutic agents including doxorubicin, cisplatin, carmustine, etoposide and cyanomorpholinodoxorubicin (MRA-CN) [48], indicating that ES-2 cells may have better clinical consequences than other CCC derived cell lines. Our finding that the ES-2 cell line was weakly positive for SHBG may imply a potential clinical role of SHBG in ovarian carcinoma although functional SHBG studies in consideration of this are warranted.

Statistical analyses of histological subtype, histological grade and FIGO stage confirmed increased SHBG expression in more unfavorable ovarian carcinomas. Moreover, SHBG is expressed significantly higher at the invasive front, known for epithelial–mesenchymal transition or transformation (EMT) processes [49]. Here tumor budding (TB) takes place, detachment and migration of small clusters of tumor cells from the neoplastic epithelium, of importance for the local invasion and distant metastases [50], [51]. This demonstrated that the local produced SHBG in ovarian cancer cells might probably influence the proliferative activity, invasiveness and distant metastasis of human ovarian carcinoma cells. Thus, our findings are consistent with the previous reports on the potential role of intracellular SHBG in other hormone-targeting carcinomas [20], [45].

Intriguingly, in salmonids the classic SHBG (SHBGa) mRNA was reported to be mainly expressed in liver and spleen, but not detected in the ovary, while a novel SHBGb had a predominant ovarian expression but could not be detected in liver [5]. At least six transcript variants of SHBG gene have been found in humans, and four of them can encode different protein isoforms (http://www.ncbi.nlm.nih.gov/gene/6462#). Both genetic factors and environment factors may contribute to the multiplicity of SHBG [52]. These variants may play an important role in some common human diseases [52], including polycystic ovary syndrome (PCOS) [53] and some cancers [54], [55]. The genetic variation in SHBG was implicated to influence prostate cancer susceptibility [54]. It is currently unknown about SHBG variants in normal and malignant ovarian tissues. Additional characterization of SHBG variants may help to further understand the molecular and biological roles of SHBG in ovarian carcinoma.

In summary, we have found that SHBG is expressed in ovarian cancer cells, verified both in cell lines and clinical samples, and its expression is associated with unfavorable clinicopathological features. The SHBG variants and their potential molecular and biological functions in human ovarian carcinoma are currently under investigation in our laboratory.
